# Over-Expression of Phosphoserine Aminotransferase-Encoding Gene (*AtPSAT1*) Prompts Starch Accumulation in *L. turionifera* under Nitrogen Starvation

**DOI:** 10.3390/ijms231911563

**Published:** 2022-09-30

**Authors:** Lei Wang, Shuiling Li, Ling Sun, Yana Tong, Lin Yang, Yerong Zhu, Yong Wang

**Affiliations:** 1 College of Life Science, Nankai University, Tianjin 300071, China; 2 Tianjin Academy of Agricultural Sciences, Tianjin 300192, China; 3 Tianjin Key Laboratory of Animal and Plant Resistance, College of Life Sciences, Tianjin Normal University, Tianjin 300387, China

**Keywords:** starch accumulation, PSAT over-expression, nitrogen starvation, *Lemna turionifera* 5511

## Abstract

It has been demonstrated that the phosphorylation pathway of L-serine (Ser) biosynthesis (PPSB) is very important in plant growth and development, but whether and how PPSB affects nitrogen metabolism and starch accumulation has not been fully elucidated. In this study, we took the energy plant duckweed (strain *Lemna turionifera* 5511) as the research object and used a stable genetic transformation system to heterologously over-expressing Arabidopsis *AtPSAT1* (the gene encoding phosphoserine aminotransferase, the second enzyme of PPSB). Our results showed that, under nitrogen starvation, the transgenic plants grew faster, with higher values of Fv/Fm, rETR, and Y(II), as well as fresh and dry weight, than the wild-type. More promisingly, the accumulation of starch was also found to be significantly improved when over-expressing *AtPSAT1* in the transgenic plants. qRT-PCR analysis results showed that the expression of genes related to nitrogen assimilation, carbon metabolism, and starch biosynthesis was up-regulated, while the expression of starch degradation-related genes was down-regulated by *AtPSAT1* over-expression. We propose that the increased starch accumulation caused by *AtPSAT1* over-expression may result from both elevated photosynthetic capacity and nitrogen utilization efficiency. This research sheds new light on the mechanism underlying the ability of PPSB to coordinate nitrogen and carbon metabolism, and provides a feasible way to improve starch production, that is, through engineering PPSB in crops.

## 1. Introduction

Duckweeds are among the smallest aquatic flowering plants, consisting of only a leaflike structure (called the frond), either with one to several pseudo-roots or without roots. Considering their high multiplication rate, high starch content, and various other practical characteristics, they have been recognized as promising biofuel plants for the generation of hydrogen, ethanol, butanol, and biogas [1,2,3]. To date, many investigations have been carried out to improve the production of starch in duckweed fronds through the alteration of cultivation conditions, including temperature, light, hormones, supplication of plant growth regulators, nutritional starvation, or abiotic stresses [4,5,6,7,8,9,10,11,12,13,14,15]. Nitrogen starvation has been shown to be one of the key factors triggering the accumulation of starch in the fronds [5,16,17].

Phosphoserine aminotransferase (PSAT; EC:2.6.1.52) is the second enzyme in the so-called phosphorylated pathway of serine biosynthesis (PPSB), which synthesizes serine from 3-phosphoglyceric acid (3-PGA) in plastids. 3-PGA is converted to Ser in three steps, sequentially catalyzed by phosphoglycerate dehydrogenase (PGDH), PSAT, and phosphoserine phosphatase (PSP). PSAT plays a key role in the biosynthesis of serine in plants and other organisms [18,19], which is one of the endogenous proteinogenic amino acids, serving as a precursor for the synthesis of glycine, tryptophan, and cysteine; as a major source of one-carbon units, which are essential for the methylation of nucleic acids and proteins; and also as an important intermediate in the biosynthesis of phospholipids, porphyrins, and nucleobases [20]. PSAT catalyzes the transamination reaction turning 3-phosphate hydroxypyruvate (3-PHP) to 3-phosphoserine, using L-glutamic acid as the amino donor accompanied by the release of 2-oxoglutamic acid [18]. Plants with reduced PPSB activity tend to exhibit a strong shoot and root growth retardation phenotype, or a lethal phenotype due to disturbed male gametophyte and embryo development. Plastid glycolysis provides the 3-PGA for PPSB, and defective PPSB can disrupt the entire carbon metabolism in plants [21]. PPSB can provide 2-oxoglutarate for glutamate synthesis and subsequent ammonium fixation (by the GS/GOGAT cycle), as well as participating in the TCA cycle through 2-oxoglutarate [22]. Previous studies on the function of PPSB in Arabidopsis have shown that it is essential for plant growth and development, linking carbon metabolism with nitrogen metabolism in the cell [18,23,24]. However, the importance of this pathway—more exactly, the PSAT—on nitrogen metabolism and starch accumulation requires further experimental verification. In Arabidopsis, two genes (*AtPSAT1* and *AtPSAT2*) have been identified which encode PSAT [25,26,27]. In PSAT1-silenced lines, the morphological characteristics showed a strong inhibition of shoot and root growth, as well as affecting metabolic pathways such as glycolysis, the tricarboxylic acid cycle (TCA), and amino acid biosynthesis [22].

In this paper, we report the stable transformation and over-expression of *AtPSAT1* in *Lemna turionifera* 5511, providing solid evidence that PSAT/PPSB regulates the vegetative growth and accumulation of starch in duckweed fronds, at least under nitrogen starvation conditions.

## 2. Results

### 2.1. Generation of AtPSAT1 Over-Expressing Duckweed

**Figure 1:**
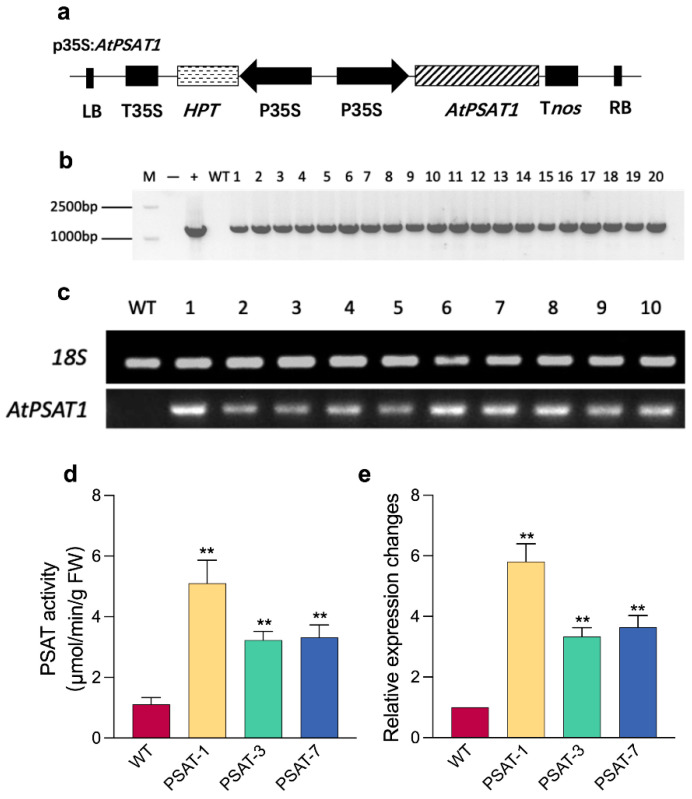
Generation and verification of *AtPSAT1* over-expressors: (**a**) T-DNA constructs designed for over-expression of *AtPSAT1*. LB, left border; HPT, encoding Hygromycin resistance gene; T35S and P35S, Terminator and Promoter of 35S, respectively; T*nos*, Terminator of *NOS*; RB, right border; (**b**) Genomic DNA-PCR analysis of *AtPSAT1* transformation. *AtPSAT1* CDs was 1293 bp. “M”, 15,000 bp DNA Ladder; “-”, negative control, water as the PCR template; “+”, positive control, plastid as the PCR template; (**c**) Semi-quantitative RT-PCR analysis of *AtPSAT1* transcripts with *18S* as reference gene; (**d**) Analysis of PSAT enzyme activity; (**e**) Real-time quantitative PCR analysis of *AtPSAT1* transcript with *18S* as reference gene. WT, wild-type; PSAT-1, PSAT-3, and PSAT-7, the three different *AtPSAT1* over-expressing lines. Values given in (**d**,**e**) are mean ± standard error (n = 3). Double asterisk symbol (**) indicates that the difference, when compared to the control, is significant based on one-way ANOVA (*p* < 0.01).

PSAT is the second key enzyme of PPSB, and is encoded by two genes—*AtPSAT1* and *AtPSAT2*—in Arabidopsis [25,26]. To explore the role of this pathway on starch accumulation and the interaction between PPSB and nitrogen assimilation in duckweed, we constructed an *AtPSAT1* over-expression vector based on pCAMBIA 1301, replacing the GUS coding sequence with the full-length CDS sequence (1293 bp) of *AtPSAT1* from Arabidopsis (Figure 1a). The vector was transformed into *Lemna turionifera* 5511 by *Agrobacterium tumefaciens* EHA105. The regenerated plants were screened with hygromycin to obtain resistant transgenic plants, and 20 transgenic lines with *AtPSAT1* over-expression were confirmed by the PCR amplification of *AtPSAT1* CDs (Figure 1b). Furthermore, 10 of these lines were analyzed for semi-quantitative RT-PCR of the *AtPSAT1* gene (Figure 1c), and three differentially expressed plants (Line 1/3/7, named PSAT-1/PSAT-3/PSAT-7, respectively) were selected for PSAT enzyme activity and quantitative RT-PCR assays. Higher PSAT enzyme activity and higher *AtPSAT1* mRNA levels were found in transgenic duckweed (Figure 1d,e).

### 2.2. Over-Expression of AtPSAT1 Affects Frond and Root of Duckweed under Datko and Nitrogen Starvation

**Figure 2:**
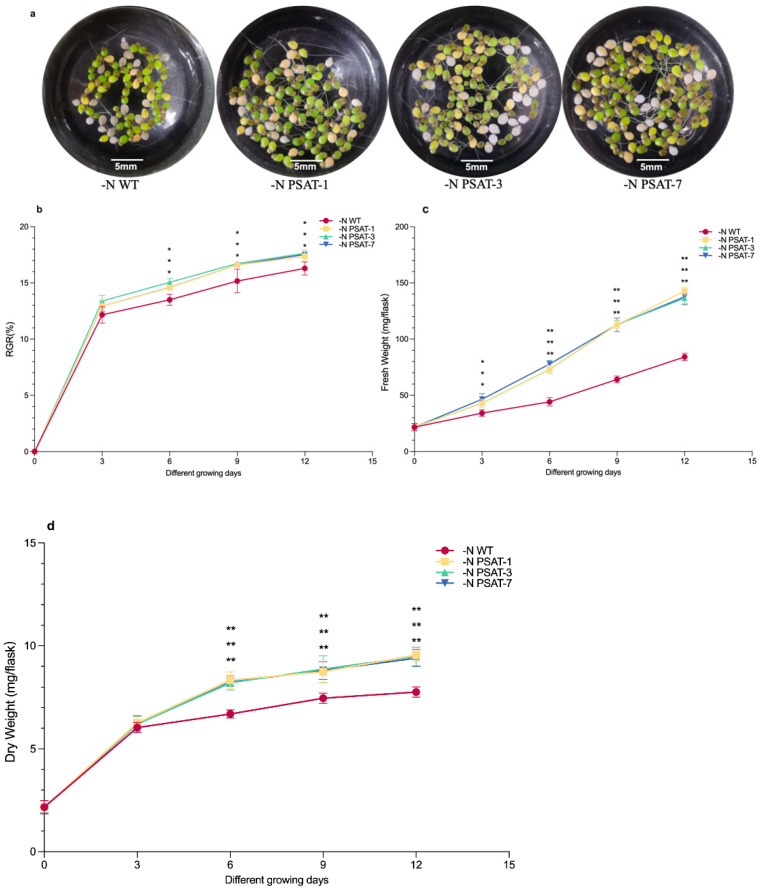

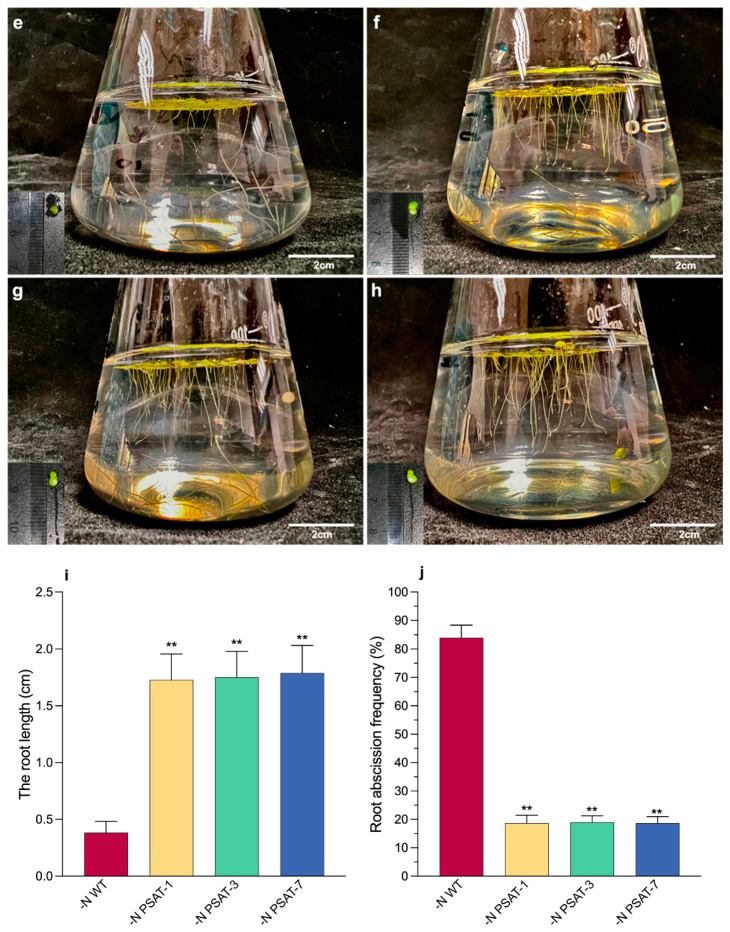
Analysis of phenotype, biomass, root length, and root abscission frequency under nitrogen starvation: (**a**) Phenotypes of WT and three *AtPSAT1* transgenic lines. Photos taken after WT and *AtPSAT1* transgenic plants were cultivated under nitrogen starvation conditions for 9 days. (**b**) Analysis of relative growth rate. (**c**) Comparison of fresh weight. (**d**) Comparison of dry weight. The relative growth rate, fresh weight, and dry weight were measured after the samples were cultivated separately under nitrogen starvation for 3, 6, 9, and 12 days. (**e**–**j**) Analysis of root phenotype, length, and abscission in WT and *AtPSAT1* transgenic lines after culturing under nitrogen starvation for 9 days. (**e**–**h**) Phenotypes of WT, PSAT-1, PSAT-3, and PSAT-7. (**i**) Comparison of root length. (**j**) Comparison of root abscission frequency. Values given are mean ± standard error (n = 3). The asterisk symbol (*) represents statistically significant differences (*p* < 0.05); the double asterisk symbol (**) indicates that the difference, compared to the control, was significant based on one-way ANOVA (*p* < 0.01).

In recent years, studies have shown that nitrogen limitation is one of the nutritional restriction factors that contribute to the accumulation of starch in duckweed [16,17,28]. *AtPSAT1* has been associated with nitrogen metabolism in plants. Both wild-type and *AtPSAT1* transgenic lines were cultivated with the Datko medium (D) and different levels of nitrogen starvation. There were almost no differences in phenotype between transgenic lines and WT in D (Datko) after 9 days of incubation (Figure S1), while there were significant morphological changes under nitrogen starvation (Figure 2a and Figure S2).

Nitrogen starvation resulted in brown, pale leaves and long, thick roots in duckweed (Figure 2a,e–h). However, WT had smaller and fewer leaves, as well as shorter and thinner roots, when compared to the transgenic lines (Figure 2a,e–h). In addition, the growth rate of the transgenic lines was significantly better than WT (Figure 2b), and the fresh and dry weights were also significantly higher than WT, when incubated under nitrogen starvation in 3d cultures (Figure 2c,d). The root length of transgenic plants was significantly longer than that of WT (Figure 2e–i), while the root shedding rate was significantly lower than that of WT (Figure 2e–j).

### 2.3. Over-Expression of AtPSAT1 Affects Photosynthetic Pigment and Chlorophyll Fluorescence-Related Parameters under Nitrogen Starvation

**Figure 3:**
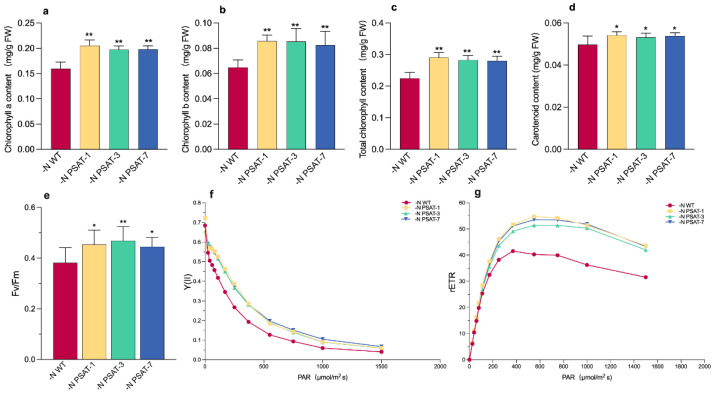
Analysis of photosynthetic pigment- and chlorophyll fluorescence-related parameters in duckweed under nitrogen starvation: (**a**) Content of chlorophyll a. (**b**) Content of chlorophyll b. (**c**) Total chlorophyll content. (**d**) Carotenoid content. The photosynthetic pigment contents were measured after the samples were cultivated under nitrogen starvation for 9 days. (**e**) Value of Fv/Fm. (**f**) Value of rETR. (**g**) Value of Y(II). Chlorophyll fluorescence-related parameters were measured after the samples were cultivated under nitrogen starvation for 6 days. Values given are mean ± standard error (n = 3) (no average values in (**f**,**g**)). The asterisk symbol (*) represents statistically significant differences (*p* < 0.05); the double asterisk symbol (**) indicates that the difference, when compared to the control, is significant based on one-way ANOVA (*p* < 0.01).

The photosynthetic pigments (chlorophylls and carotenoids) of WT and transgenic lines were measured in the samples treated under nitrogen starvation and full-nutrient conditions (Datko medium; D) for 9 days. There was no difference between WT and transgenic duckweed under Datko conditions (Figure S3a–d). Meanwhile, compared with the control, we found that the contents of chlorophyll a (Figure 3a), chlorophyll b (Figure 3b), and total chlorophyll (Figure 3c) were significantly higher under nitrogen starvation in transgenic lines. The abundance of carotenoids was also significantly higher in transgenic lines than WT under nitrogen starvation (Figure 3d). In order to verify whether *AtPSAT1* is involved in the protection of photosynthesis capability under nitrogen starvation, several chlorophyll fluorescence-related parameters were subsequently measured after 6 days of nitrogen starvation or Datko treatment. Both WT and transgenic lines showed a similar value for chlorophyll fluorescence-related parameters (Figure S3e–g). Interestingly, we observed that Fv/Fm (PSII maximum light quantum production) was 10% higher in transgenic lines, compared with WT, after nitrogen starvation (Figure 3e). rETR (relative electron transfer rate) and Y(II) (PS II actual photosynthetic efficiency) were also higher in transgenic lines than WT under nitrogen starvation (Figure 3f,g).

### 2.4. Over-Expression of AtPSAT1 Affects Starch, Sugar, and Soluble Protein Contents under Nitrogen Starvation

**Figure 4:**
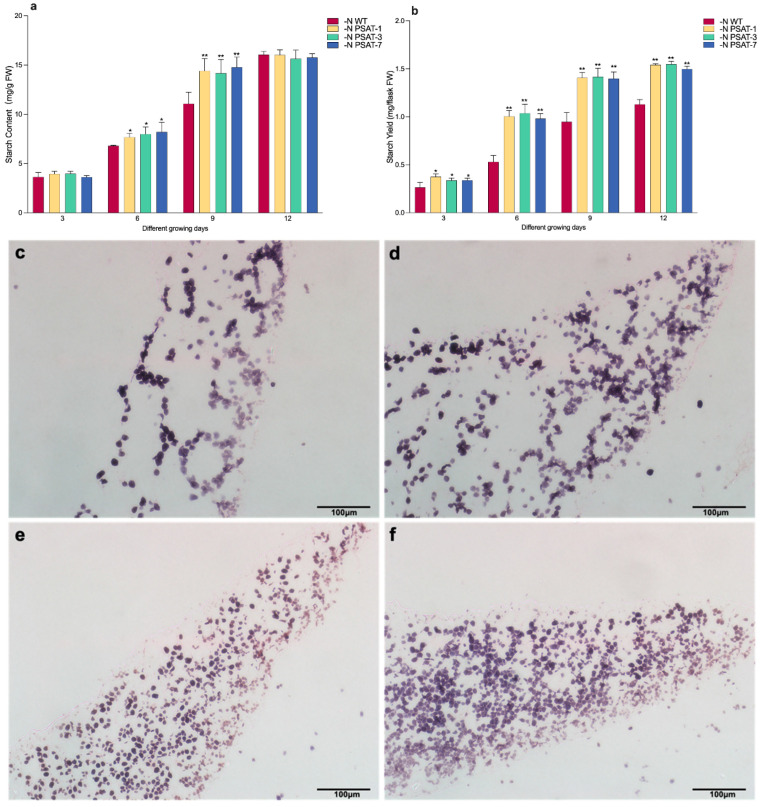

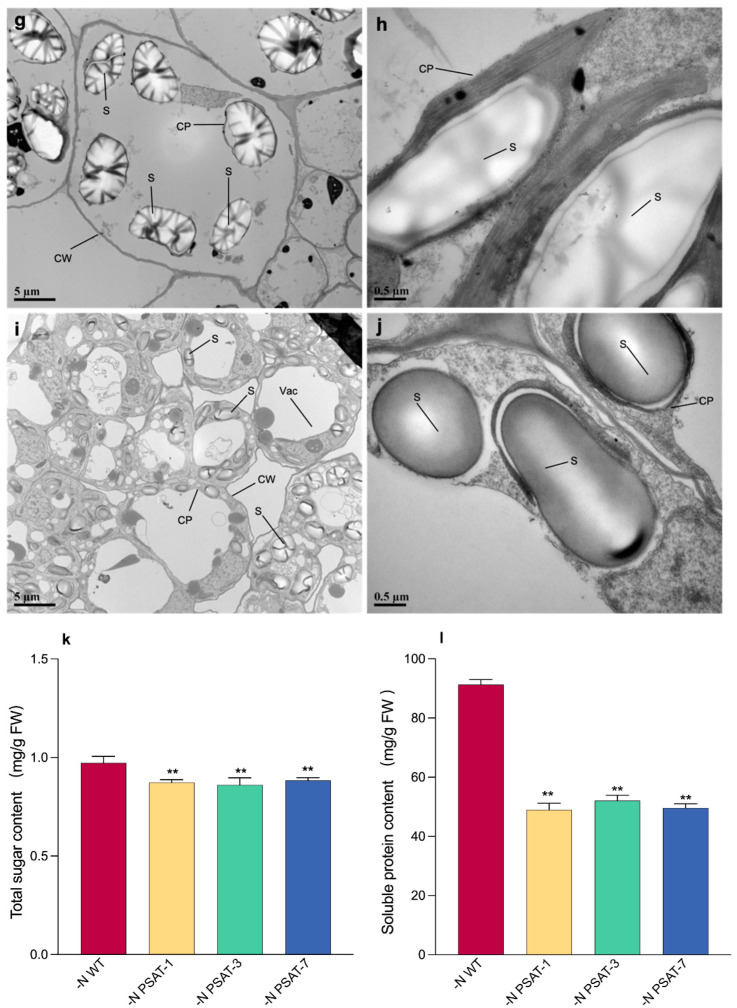
Changes in the content of starch, sugar, protein, cellular level morphology, and structure in duckweed after nitrogen starvation for 9 days: (**a**) Starch content. (**b**) Starch yield. (**c**,**d**,**g**,**h**) The cellular level morphology and structure of WT. (**e**,**f**,**i**,**j**) The cellular level morphology and structure of PSAT-7. CW, cell wall; S, starch; Vac, vacuole; CP, chloroplast. (**k**) Total sugar content. (l) Soluble protein content. Values given in (**a**,**b**,**k**,**l**) are mean ± standard error (n = 3). The asterisk symbol (*) represents statistically significant differences (*p* < 0.05), and the double asterisk symbol (**) indicates highly significant differences (*p* < 0.01) based on one-way ANOVA.

The starch content and yield were measured under Datko conditions (D) and different nitrogen content culture conditions (Figures S4–S6 and Figure 4a,b). The results indicated that, under full-nutrient Datko conditions, there were no significant differences in starch content and starch yield between WT and transgenic lines after 3, 6, 9, and 12 days of cultivation, although there was a slight increase, from about 1.3 to 2.5 mg starch per g fresh weight, and 0.04 to 0.75 mg starch per flask (Figure S4). Similar results were found for the different samples, with their starch content showing almost no significant differences under shortage of 2/5 nitrogen (−2/5N) or 4/5 nitrogen (−4/5N) at days 3, 6, 9, and 12 (Figures S5 and S6). However, the transgenic lines showed significantly higher starch content and starch yield than WT under nitrogen starvation (−N) culture conditions at days 6 and 9 during the 12 days of cultivation (Figure 4a,b, Figures S5 and S6). Nitrogen starvation for 9 days led to a significant accumulation of starch. These results suggest that nitrogen starvation was more advantageous for the transgenic lines than WT, in terms of promoting starch accumulation. Therefore, we focused the remainder of the analysis on day 9 of the nitrogen starvation treatment.

I_2_-KI-stained sections of WT and PSAT-7 further illustrated starch accumulation at the cellular level (Figure 4c–f), which indicated that the cells of nitrogen starvation fronds were filled with a large number of black–purple granules (starch granules), where the transgenic lines had much more starch granules than WT. The transmission electron microscopy (TEM) observation results of chloroplasts in mesophyll cells confirmed that there were larger starch granules in WT fronds than in transgenic lines after 9 days of nitrogen starvation (Figure 4g–j). Furthermore, the number of starch granules in the transgenic lines PSAT-7 seemed significantly higher than that in WT under the same observation field. These results re-confirmed that starch accumulation was higher in the transgenic lines than in the WT under nitrogen starvation at day 9.

The dry weight and starch content of transgenic plants were all higher than that of the control under nitrogen starvation (Figure 2d and Figure 4a). The dry weight is composed mainly of carbohydrates and proteins, as there is little fiber and lipid content in duckweed. The total sugar and soluble protein content were analyzed under nitrogen starvation (Figure 4). We found that nitrogen starvation significantly affected the total sugar content and soluble protein content of duckweed (Figure 4k,i): The contents of total sugar and soluble protein were decreased in the *AtPSAT1* lines under nitrogen starvation, especially the protein content (Figure 4i).

### 2.5. Over-Expression of AtPSAT1 Affects Enzyme Activity of Nitrogen Assimilation and Expression of Nitrogen Assimilation-Related Genes under Nitrogen Starvation

**Figure 5:**
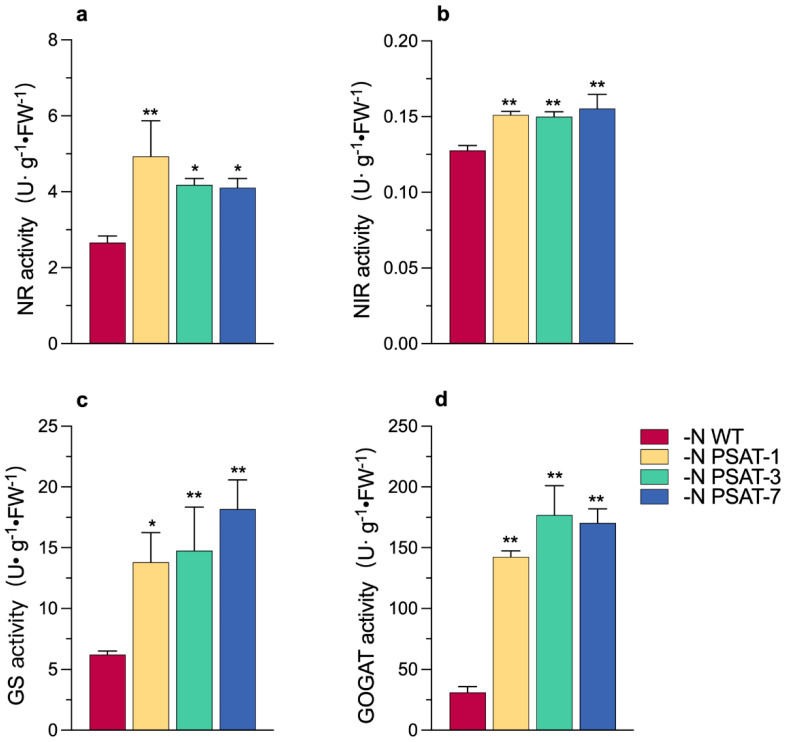
Analysis of enzyme activity involved in nitrogen assimilation in duckweed after nitrogen starvation for 6 days: (**a**) Nitrate reductase activity; (**b**) Nitrite reductase activity; (**c**) Glutamine synthetase activity; and (**d**) Glutamate synthase activity. Values given are mean ± standard error (n = 3). The asterisk symbol (*) represents statistically significant differences (*p* < 0.05), and the double asterisk symbol (**) indicates highly significant differences (*p* < 0.01) according to one-way ANOVA.

**Figure 6:**
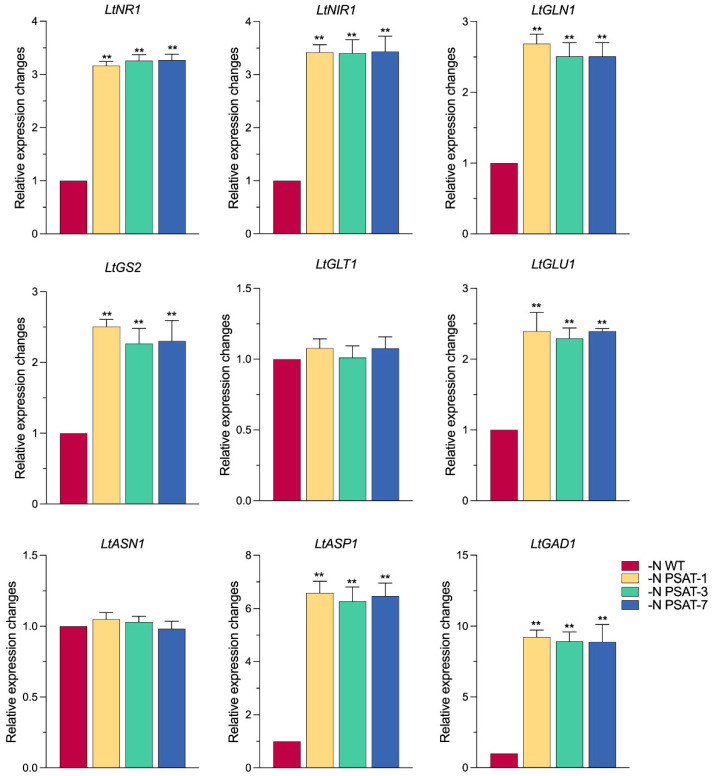
Relative expression levels of gene related to nitrogen assimilation in duckweeds after nitrogen starvation for 6 days. The relative expression level of the WT was normalized to 1. Values given are mean ± standard error (n = 3). The double asterisk symbol (**) indicates highly significant differences (*p* < 0.01) according to one-way ANOVA.

The above results suggested that the growth in over-expression *AtPSAT1* lines was better than that of the wild-type, and the increasing dry weight in the transgenic lines was mainly due to starch accumulation under nitrogen starvation, while nitrogen assimilation is closely related to plant growth and development [29]. How are growth and starch synthesis maintained in *AtPSAT1* over-expression lines under nitrogen starvation? First, we measured the enzyme activities of NR (nitrate reductase), NIR (nitrite reductase), GS (glutamine synthetase), and GOGAT (glutamate synthase) after nitrogen starvation for 6 days (Figure 5). The NR and NIR activities of the transgenic lines were found to be significantly higher than that of the WT (Figure 5a,b). The activity of GS in transgenic lines was 2–3 times that in WT (Figure 5c). The activity of GOGAT was also significantly higher than that in WT, by 5–6-fold (Figure 5d). Then, we measured the expression patterns of genes encoding the enzymes NR, NIR, ASN (asparagine synthetase), ASP (aspartate aminotransferase), GAD (glutamate decarboxylase), GS, and GOGAT, in order to analyze the effect of nitrogen starvation on nitrogen assimilation in transgenic duckweed (Figure 6). The results showed that, after 6 days of nitrogen starvation, the expression of all genes was significantly up-regulated in the transgenic lines, compared to WT, except for *LtASN1* and *LtGLT1*, for which no difference between the WT and transgenic lines was observed. The expression of *LtNIR1* and *LtNR1* were both significantly up-regulated (approximately 2.8-fold and 3.0-fold, respectively), *LtASP1* was up-regulated 5-fold on average, *LtGAD1* was up-regulated close to 10-fold on average, and *LtGLN1* and *LtGS1* encoding GS were significantly up-regulated 2.6-fold and 2.5-fold on average, respectively. The expression pattern of *LtGLT1* encoding GOGAT was up-regulated but not significantly different, and *LtGLU1* was up-regulated approximately 2-fold, with PSAT-1 being the most significant.

### 2.6. Over-Expression of AtPSAT1 Affects Starch Metabolism Genes under Nitrogen Starvation

**Figure 7:**
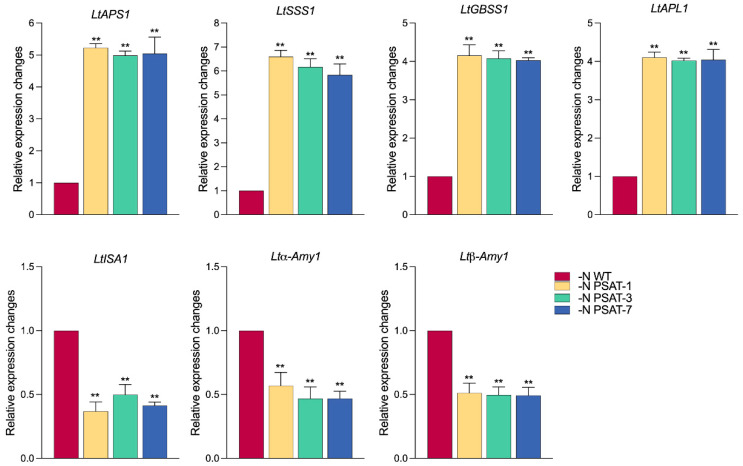
Relative expression levels of genes related to starch synthesis and metabolism in duckweed after nitrogen starvation for 6 days. The relative expression level in WT was normalized to 1. Values given are mean ± standard error (n = 3). The double asterisk symbol (**) indicates highly significant differences (*p* < 0.01) according to one-way ANOVA.

Starch biosynthesis involves a series of key enzymes, including ADP-glucose pyrophosphorylase (AGP), soluble starch synthase (SSS), and granule-bound starch synthase (GBSS). Starch degradation is mainly driven by isoamylase (ISA), α-amylase (α-Amy), and β-amylase (β-Amy) [30]. To investigate the gene expression patterns of transcripts encoding these key enzymes, we analyzed their relative expression levels through qRT-PCR (Figure 7). AGP is a regulator of the first step of starch biosynthesis. It consists of two large sub-units (APL), which have regulatory function, and two small sub-units (APS), which are responsible for the catalytic activity in plants [31,32]. The results showed that the expression of *LtAPS1* was significantly higher in the transgenic lines than WT, with a 5.5-fold increase in nitrogen starvation, while the expression of *LtAPL1* did not significantly differ between the transgenic lines and WT. The key enzymes GBSS and SSS (encoded by *LtGBSS1* and *LtSSS1*, respectively) in starch synthesis provide the glucose substrate for straight-chain starch elongation and transfer glucose units to straight-chain starch molecules, respectively [33,34,35]. The gene encoding SSS, *LtSSS1,* also showed a different expression pattern, with significantly higher expression in the transgenic lines than WT, with the highest expression of PSAT-1 among the three transgenic materials, increasing 6.5-fold compared to WT (Figure 7). The expression of *LtGBSS1*-encoded GBSS presented similar results. The expression of *LtGBSS1* in transgenic lines was significantly higher than that in WT, with the highest expression of PSAT-1 being 4-fold higher than in WT (Figure 7). α-amylase is the key enzyme for endo-hydrolysis of the α-1,4 glucosyl bond, whereas β-amylase is considered to be the main hydrolytic enzyme for breaking down starch granules [33]. The expression of *LtISA1*, encoding isoamylase, was significantly lower than in WT, by about 0.5-fold, in all transgenic lines; both α- and β-amylase, responsible for degrading starch into smaller hydrocarbons, were down-regulated in the transgenic lines (Figure 7). The expression of *Ltα-Amy1*, which encodes α-amylase, was reduced by about 0.5-fold on average, while the highest expression of *Ltβ-Amy1*, which encodes β-amylase, was only half that of WT in the transgenic lines (Figure 7).

### 2.7. Over-Expression of AtPSAT1 Affects Carbon Metabolism under Nitrogen Starvation

**Figure 8:**
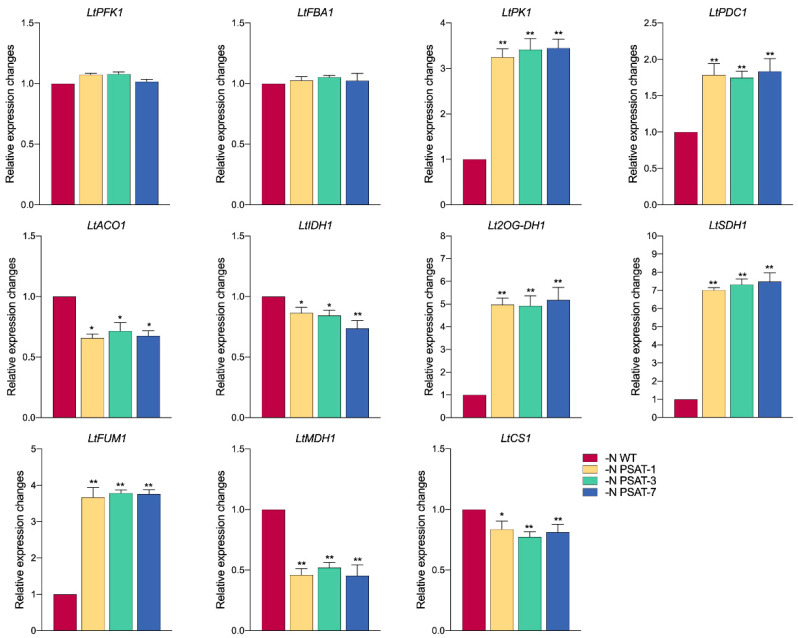
Relative expression levels of genes related to carbon metabolism in duckweed after nitrogen starvation for 6 days. The relative expression level in the WT was normalized to 1. Values given are means ± standard errors (n = 3). The asterisk symbol (*) represents statistically significant differences (*p* < 0.05), and the double asterisk symbol (**) indicates highly significant differences (*p* < 0.01) according to one-way ANOVA.

Starch degradation and biosynthesis are closely linked to the glycolytic pathway, involved in the carbon cycle process from starch–glucose–starch. In plastids, starch is metabolized by hydrolysis to produce glucose, thus participating in the glycolytic pathway [36]. We investigated the expression of genes encoding several key enzymes in the glycolytic pathway. The results demonstrated that the expression of *LtPFK1* (phosphofructokinase) and *LtFBA1* (fructose-bisphosphate aldolase) did not differ significantly between the transgenic lines and WT after nitrogen starvation for 6 days, while the expression of *LtPK1* (pyruvate kinase) was significantly higher in transgenic lines than in WT by about 3-fold, and *LtPDC1* (pyruvate dehydrogenase complex) expression was also significantly higher than WT, by about 1.7-fold (Figure 8).

The TCA (the tricarboxylic acid) cycle is a common oxidation pathway for the sugars, lipids, and proteins, which also has some association with the glycolytic pathway [16]. In addition, 2-ketoglutarate, which is the metabolite of PSAT, is also involved in the TCA cycle through anaplerotic reaction [18]. After 6 days of nitrogen starvation, we found that the expression patterns of key enzyme genes involved in the TCA cycle presented differently. Compared with the WT, the expression levels of *LtACO1* (aconitase), *LtIDH1* (isocitrate dehydrogenase), *LtMDH1* (malate dehydrogenase), and *LtCS1* (citrate synthase) were significantly down-regulated, while *Lt2OG-DH1* (2-OG dehydrogenase), *LtSDH1* (succinate dehydrogenase), and *LtFUM1* (fumarase) were significantly up-regulated (Figure 8) in transgenic duckweed.

## 3. Discussion

### 3.1. Over-Expression of AtPSAT1 Prompts Growth by Regulating Utilization of Endogenous Nitrogen under Nitrogen Starvation

The effective utilization of nitrogen, a key element for life, has a great impact not only on plant growth and development but also on the biomass and productivity of the plant [37]. The results of our study demonstrated that there was almost no difference between WT and the *AtPSAT1* over-expression lines in duckweed under normal conditions; however, under nitrogen starvation conditions, the relative growth rate, fresh weight, and dry weight in *AtPSAT1* over-expression of *L. turionifera* were significantly higher than those of the WT (Figure 2). The observed increase in dry weight was in agreement with reports in *L. punctata* 0202 under nitrogen starvation conditions. They concluded that endogenous nitrogen may support growth under nitrogen starvation conditions for a while, as duckweed is rich in endogenous nitrogen [17]. PSAT is responsible for the second catalytic step in the phosphorylated serine (PPSB) pathway, which is known for its involvement in the processes of plant growth and development [18,20]. Recently, the PPSB pathway has been suggested to play the role of a link between plant growth and nitrogen metabolism [18,20]. These findings suggest that *AtPSAT1* over-expression could serve to maintain the growth of transgenic lines for a while, through the use of endogenous nitrogen under nitrogen starvation conditions.

To verify the above speculation, we performed qRT-PCR and enzyme activity analyses regarding the key genes and enzymes related to nitrogen assimilation and utilization. There are several key enzymes responsible for the absorption, reduction, and transportation of nitrogen, as well as the transamination reaction in *L. turionifera*, such as nitrate reductase (NR, encoded by *LtNR1*), nitrite reductase (NIR, encoded by *LtNIR1*), glutamine synthetase (GS, encoded by *LtGLN1* and *LtGS2*), and glutamate synthase (GOGAT, encoded by *LtGLU1*). The transcription profiling of these key genes, as well as the activities of their corresponding enzymes, were all promoted in *AtPSAT1* over-expressing transgenic lines, compared with WT, under nitrogen starvation conditions (Figure 5 and Figure 6). It seems that *AtPSAT1* over-expression enhanced the reduction of endogenous nitrogen nitrate, producing ammonia by up-regulating the expression of *LtNR1* and *LtNIR1,* as well as enhancing the enzyme activities of NR and NIR, as nitrate absorbed is mainly reduced to ammonium by NR and NIR in plants [37], followed by ammonia assimilation. The assimilation process was likely prompted, generating glutamine by GS catalysis, due to the high expression of *LtGLN1* and *LtGS2,* as well as increased GS activity in transgenic lines. This is evidenced by the enzyme activities of GS and GOGAT being increased 3–4- and 5–7-fold, respectively, in comparison with the WT. It has been reported that GS has a higher affinity for ammonium [38], with approximately 95% of ammonium being assimilated through the GS–GOGAT cycle [29]. In addition to the major ammonium assimilation, the GS–GOGAT pathway plays a key role in ammonium re-assimilation [39]. The glutamine produced by GS was likely converted to other amino acids quickly, such as glutamate catalyzed by GOGAT, aspartate catalyzed by ASP, and GABA catalyzed by GAD, as the expression levels of these genes were up-regulated by approximately 2-, 5-, and 10-fold, respectively. These amino acids all play important roles in plants. Glutamate acts as the main intra- and inter-cellular nitrogen carrier [40,41], which demonstrates that the over-expression of *AtPSAT1* improved endogenous nitrogen transport and re-use. GS is regarded as one of the most important factors for nitrogen use efficiency (NUE) [42]. The high GS activity caused by *AtPSAT1* over-expression would be helpful in increasing the NUE in transgenics lines. Meanwhile, the lower content of protein in *AtPSAT1* transgenic lines suggested that, under nitrogen deficiency, the transgenic duckweed probably met the nitrogen source requirement for its growth by increasing endogenous protein degradation (Figure 4l), confirming that over-expression of *AtPSAT1* promoted growth by up-regulating the expression of genes related to endogenous nitrogen recycling and ammonium assimilation in duckweed.

Fv/Fm denotes the maximum quantum efficiency of photosynthetic tissue and is an important indicator of plant photosynthetic activity. Generally, Fv/Fm values are about 0.8 in healthy plants. Both for the wild-type and transgenic duckweeds, their value of Fv/Fm was above 0.8 when they were cultured in the Datko medium, suggesting that they were healthy under full-nutrition conditions (Figure S3). When the Fv/Fm value is lower than 0.8 in photosynthetic organisms, it indicates that the plant is subject to some environmental stress [43]. Fv/Fm values were below 0.8 under nitrogen starvation in all samples; however, it was significantly higher in *AtPSAT1* transgenic lines than in WT. Meanwhile, compared with the wild-type, the PSII-mediated relative electron transport rate (rETR) and the actual photochemical efficiency of PSII (Y(II)) were both substantially higher in the transgenic lines. This further indicated that over-expression of *AtPSAT1* is helpful for maintaining photosystem function to prompt the growth of duckweed under nitrogen starvation.

### 3.2. Over-Expression of AtPSAT1 Prompts Starch Accumulation by Regulating Starch Synthesis, Glycolysis, and TCA under Nitrogen Starvation

Starch is one of the main storage compounds in duckweed, and is usually accumulated under stress conditions (e.g., nitrogen starvation). This is an adaptive strategy acquired during long-term evolution that allows plants to survive in unfavorable environments [30]. In our study, the changes in starch content showed different trends under full-nutrient and nitrogen starvation conditions. There was almost no difference between the WT and transgenic plants under full-nutrient conditions (Figure S4); however, the starch content of the transgenic duckweed was significantly higher than that in WT under nitrogen starvation (Figure 4a,b). These results suggest that over-expression of *AtPSAT1* can effectively promote starch accumulation in duckweed under nitrogen starvation conditions.

Starch accumulation is closely related to starch synthesis and metabolism. AGPase, GBSS, and SSS are all key enzymes of the starch biosynthesis pathway in higher plants. *LtAPS1* is the gene encoding the small sub-unit of AGPase, which plays an important role in regulating starch levels and determining the starch deposition pattern of plants [44]. In our experiment, *LtAPS1*, *LtSSS1,* and *LtGBSS1* were all significantly up-regulated in the transgenic lines under nitrogen starvation (Figure 6), in agreement with the results of Yu et al. [16] and Tao et al. [13], suggesting that the high accumulation of starch was prompted by enhancement of the starch synthesis pathway in *AtPSAT1* over-expression lines under nitrogen starvation. We believe that starch accumulation is also related to the decreased starch degradation in *AtPSAT1* transgenic lines, as the total sugar content in transgenic lines was lower than that detected in WT, and the expression of three key genes involved in the starch degradation pathway was down-regulated, including isoamylase,α- and β-amylases (Figure 6). This result is consistent with the findings of Guo et al. [17]. Previous studies have shown that down-regulation of chloroplast-targeted potato β-amylase led to a starch overload phenotype [33], and the isoamylase is involved in the starch degradation process and can significantly affect the starch content [34]. Taken together, the accumulation of starch caused by *AtPSAT1* engineering resulted from the elevated activity of starch synthesis-related enzymes and decreased activity of starch metabolism-related enzymes in the transgenic lines under nitrogen starvation.

**Figure 9:**
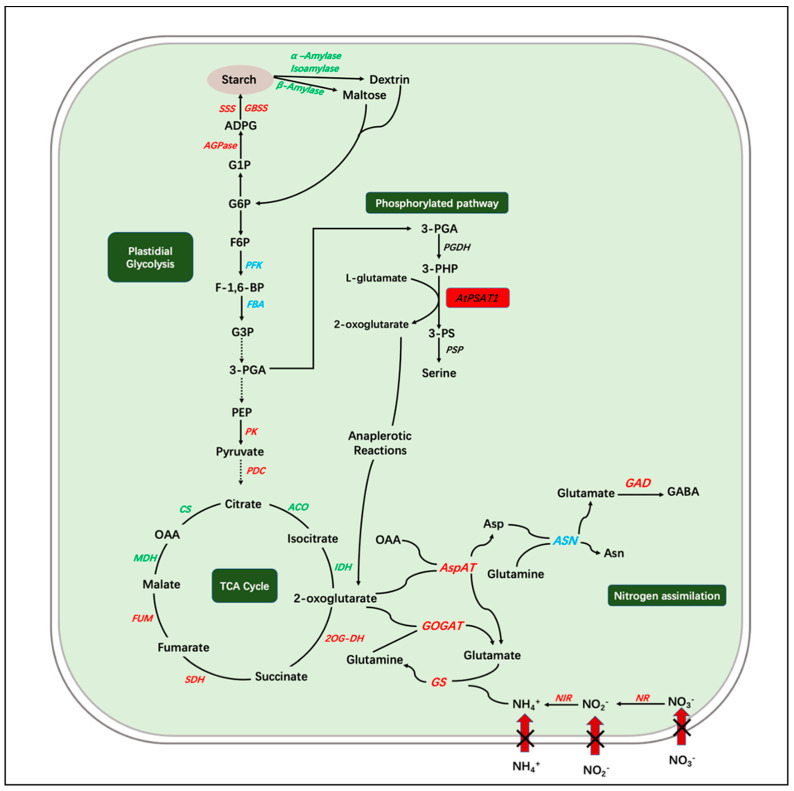
Expression pattern of genes involved in serine phosphorylated synthesis pathway, carbon metabolism, starch metabolism, and nitrogen assimilation under nitrogen starvation in transgenic duckweed. The arrows indicate the directions of catalytic reactions or transportation. Red indicates up-regulated expression, green indicates down-regulated expression, and blue indicates no significant change. Dotted black arrows indicate omitted steps. AGPase, ADP-glucose pyrophosphorylase; ADPG, adenosine-5-diphosphoglucose; GBSS, granule-bound starch synthase; SSS, soluble starch synthase; G1P, glucose 1-phosphate; G6P, glucose 6-phosphate; F-1,6-BP, fructose1,6-bisphosphatase; F6P, fructose 6-phosphate; PFK, phosphofructokinase; FBA, fructose-bisphosphate aldolase; G3P, glyceraldehyde 3-phosphate; 3-PGA, 3-phosphate glycerate; PEP, phosphoenolpyruvate; PK, pyruvate kinase; PDC, pyruvate dehydrogenase complex; OAA, oxaloacetate; 2-OG DH, 2-OG dehydrogenase; ACO, aconitase; CS, citrate synthase; FUM, fumarase; IDH, isocitrate dehydrogenase; MDH, malate dehydrogenase; SDH, succinate dehydrogenase; TCA, tricarboxylic acid; 3-PHP, 3-phosphohydroxypyruvate; 3-PS, 3-phosphoserine; PGDH, 3-phosphoglycerate dehydrogenase; *AtPSAT1*, the gene encoding 3-phosphoserine aminotransferase of Arabidopsis; PSP, 3-phosphoserine phosphatase; AspAT, aspartate aminotransferase; ASN, Asparagine synthetase; GABA, gamma-Aminobutyric acid; GOGAT, glutamate synthase; GS, glutamine synthase; Asn, asparagine; Asp, aspartate; NR, nitrate reductase; NIR, nitrite reductase.

The phosphorylated serine pathway (PPSB) is known for its role in regulating the glycolytic flux and tricarboxylic acid cycle (TCA) [45]. Glycolysis is one of the main pathways of carbon metabolism in plants, and starch is the main substrate for the initial step of glycolysis—decomposing starch through the classical intermediate metabolism of glycolysis [31,32]. The increase in starch content and the decrease in total sugar content in transgenic materials suggested that transgenic duckweed likely accumulated starch by regulating the mutual conversion between starch and sugar. The change in expression levels of key genes in the pathway of glycolysis and TCA demonstrated that PPSB is highly correlated with the process of glycolysis and TCA in *AtPSAT1* over-expression lines under nitrogen stress (Figure 9). The relative expression levels of *LtPFK1* (encoding phosphofructokinase) and *LtFBA1* (encoding fructose bisphosphate aldolase) did not significantly differ between transgenic lines and WT, while *LtPK1* (encoding pyruvate kinase) and *LtPDC1* (encoding pyruvate dehydrogenase complex) were significantly higher in transgenic lines than WT. Pyruvate kinase (PK) is a key regulatory enzyme that catalyzes the conversion of phosphoenolpyruvate (PEP) to ATP and pyruvate in the glycolytic pathway [46]. The up-regulation of *LtPK1* and *LtPDC1* in transgenic lines likely prompts the production and metabolism of pyruvate, as a carbon backbone, to increase amino acids and intermediates in the TCA cycle. Furthermore, the 2-oxoglutarate (2-OG) released by PSAT activity can be directly recycled to glutamate by the GOGAT enzyme in the plastid [45]. 2-OG produced in the PPSB seems to be the interconnection point of carbon and nitrogen metabolism. Under nitrogen starvation, over-expression of *AtPSAT1* is helpful in maintaining the metabolic balance of TCA cycle intermediates. In conclusion, the expression patterns of genes involved in TCA seem to regulate the content of 2-ketoglutarate in a coordinated manner, prompting it to enter the pathway of carbon and nitrogen metabolism, which may result in decreasing sugar content and starch accumulation after over-expression of *AtPSAT1* under nitrogen starvation.

## 4. Materials and Methods

### 4.1. Duckweed Culture and Nitrogen Starvation Treatment

*Lemna turionifera* 5511 were cultivated in aseptic Datko medium, according to Wang and Kandeler [47]. The duckweed was cultured at a temperature of 22 ± 2 °C under long-day light period conditions (16 h light/8 h dark cycle), and a light intensity of 100 μmol photons m^−2^·s^−1^. At first, fresh fronds were grown on Datko medium for 7 days. Then, 20 fronds with constant growth status were transferred to Datko medium that lacked nitrogen or fresh Datko medium in a 100 mL conical flask. The plants were cultivated in the same long-day light period conditions and temperature, with a light intensity of 100 μmol photons m^−2^·s^−1^ at the plant level.

### 4.2. Vector Construction and the Transgenic Duckweed Verification

The sequence of the known *AtPSAT1* (first encoded gene of 3-phosphoserine aminotransferase) was obtained from the *Arabidopsis thaliana* cDNA by PCR. It was inserted into the plasmid of pCAMBIA1301 with *hygromycin* resistance gene, and modified with CaMV-35S promoter at 5′ and the terminator of the nopaline synthase gene at 3′ end, in order to construct the plant binary expression vector pCAMBIA 1301-35S-*AtPSAT1*. According to the freeze–thaw method, the vector was transformed into the *Agrobacterium tumefaciens* strain EHA105. The transgenic duckweed of *AtPSAT1* was acquired using the callus transformation system described by Yang at al. [48]. DNA isolation was followed by the SDS method, also described by Yang et al. [48]; RNA was isolated using an Eastep Super Total RNA Extraction Kit (Promega, Shanghai, China) and reverse transcription to cDNA using a PrimeScript^TM^RT reagent Kit with gDNA Eraser (TaKaRa, Beijing, China). The DNA and cDNA for *Lt18s* and *AtPSAT1* were amplified using PCR and RT-PCR with specific primers (provided in Supplementary Materials, Table S1), in order to identify the transgenic duckweed.

### 4.3. Measurement of the PSAT Enzyme Activity

The PSAT activity was measured according to the method described by Ho Chai-ling and Masaaki [25], where the assay mixture contained 50 mM Tris-HCl (pH 8.2), 32 mM ammonium acetate, 2 mM glutamate, 0.2 mM NADH, 2.5 mM phosphate hydroxypyruvate, 2 units of glutamate dehydrogenase, and 10 µL of protein extract. The reaction was initiated by the addition of phosphate hydroxypyruvate at 25 °C, and the change in absorbance at 340 nm was measured. One unit of enzyme activity was defined as the amount of 1 μmol of substrate formed per minute of catalysis. The protein concentration was then determined according to Bradford’s method.

### 4.4. Measurements of Biomass and RGB

We cultured 10 duckweed plants with the same growth state in each 100 mL conical flask containing the culture solution, and weighed the fresh weight separately on different growth days. The duckweed samples treated for different days were weighed immediately after draining the water with a pump, using the fresh weight of the flask; after that, they were dried in a hot air circulating oven at 80 °C to a constant weight, and then weighed using the dry weight of the flask.

The growth rate of duckweed was calculated according to the change in fresh weight, which was weighed at the first growth day (*d*_0_ *=* 0 *d*) as the initial fresh weight (taking 10 lines of duckweed with the same growth status and taking fresh weight) and at the end of the growth day (*d_x_ =* 3 *d*, 6 *d*, 9 *d*, or 12 *d*). The relative growth rate (RGR) was calculated according to Su and Ziegler [49,50]:

RGR = (ln*Wd_x_* − ln*Wd*_0_)/(*d_x_* − *d*_0_), (1)

where ln represents the natural logarithm, and *Wd_x_* and *Wd*_0_ represent the fresh weight at *d_x_* and *d*_0_, respectively.

### 4.5. Duckweed Phenotype Observation, Root Length Measurement, and Statistics

Roots and fronds were observed and photographed using a camera and an Olympus MVX10 dissecting stereo microscope. The root lengths of 30 randomly selected lines of duckweed were measured using the ImageJ software. We also statistically analyzed the number of roots which had fallen off 30 randomly selected lines of duckweed (duckweed usually has two roots, under the mother frond and daughter frond).

### 4.6. Quantification of Photosynthetic Pigment and Chlorophyll Fluorescence Parameters

Next, 0.1 g of duckweed material treated for 9 days was immersed in 1 mL of 95% ethanol and placed in an incubator at 28 °C until the duckweed turned completely white. Then, 200 μL of the extract was taken and its OD values at the wavelengths of 663 nm, 645 nm, and 440 nm were measured under a plate reader, and the photosynthetic pigment content was calculated according to the Arnon method [51]. Chlorophyll fluorescence parameters were measured using a Mini-PAM-II (WALZ, Effeltrich, Germany). The Fv/Fm, rETR, and Y(II) of photosystem II were measured according to the WALZ protocol.

### 4.7. Observation of Frond Morphology and Ultrastructure

For observation of the morphology and ultrastructure of fronds, the duckweeds were sampled after 9 days of cultivation without nitrogen. For I_2_-KI-stained sections, fronds were prepared in paraffin sections. According to the method described by Zhu [12], the sections were stained with I_2_-KI staining solution for 5–10 min and the slices were sealed directly with staining solution for observation and comparison of tissue structure by light microscopy. For TEM samples, plant tissues were collected and fixed overnight with 2.5% glutaraldehyde solution at 4 °C. The fixed solution was poured out, rinsing the sample with 0.1 M pH 7.0 phosphoric acid buffer three times, each time for 15 min. The samples were fixed with 1% osmium solution for 1–2 h. Osmium acid waste liquid was carefully taken out, again rinsing the sample with 0.1 M pH 7.0 phosphoric acid buffer three times, each time for 15 min. The samples were dehydrated with a gradient of ethanol solutions (30%, 50%, 70%, 80%, 90%, and 95%). Each concentration was treated for 15 min, after which it was treated with 100% ethanol twice for 20 min and finally treated with pure acetone for 20 min. The mixture of embedding and acetone (*v*/*v* = 1/1) was used to treat the sample for 1 h, then another mixture of embedment and acetone (*v*/*v* = 3/1) was used to treat the sample for 3 h. The sample treated with pure embedding agent was covered overnight. The embedded sample was then embedded and heated at 70 °C overnight, then sliced using a Leica EM uc 7 ultra-thin microtome, with which 70–90 nm sections were obtained. The sections were stained with a lead citrate solution and 50% alcohol saturated solution of uranyl acetate for 5–10 min, then observed using a HITACHI H-7650 (HITACHI, Tokyo, Japan).

### 4.8. Measurement of Starch, Total Sugar, and Soluble Protein Contents

A starch extraction and measurement kit was used to extract and measure the starch content, according to the manufacturer’s protocol (BC0700, Solarbio Biological & Technology Co. Ltd., Beijing, China). The total sugar was extracted and measured using a total sugar extraction and measurement kit, according to the manufacturer’s protocol (BC2715, Solarbio Biological & Technology Co. Ltd., Beijing, China). Soluble protein content extraction was carried out using 30 mM Tris-HCl (pH 7.0), and was measured using the manufacturer’s protocol (P0006C, Beyotime Biotechnology Co. Ltd., Shanghai, China).

### 4.9. Measurement of NR, NIR, GS, and GOGAT Activities

A nitrate reductase (NR), nitrite reductase (NIR), glutamine synthetase (GS), and glutamate synthase (GOGAT) extraction and measurement kit was used to extract and measure the enzyme activities, according to the manufacturer’s protocol (Solarbio Biological & Technology Co., Ltd., Beijing, China).

### 4.10. qRT-PCR Analysis of Gene Expression

RNA was isolated and reverse transcribed to cDNA; the cDNA for *Lt18s*, *AtPSAT1*, starch metabolism genes, nitrogen assimilation genes, and carbon metabolism genes was amplified using qRT-PCR with specific primers, designed from the duckweed genome sequence obtained by RNA-Seq (provided in Table S1). qRT-PCR was performed using an iCycler Thermal Cycler (Bio-Rad iQ5, Hercules, CA, USA) with TB Green Premix Ex TaqII (Code No. RR420A:TB Green Premix Ex Taq TM, Beijing Takara, Beijing, China), according to the manufacturer’s protocol. The reaction mixture was heated to 95 °C for 30 s, followed by 40 PCR cycles at 95 °C for 5 s, 58 °C for 30 s, and 72 °C for 30 s. The differences in the relative expression levels of detected genes were calculated using the 2^−ΔΔCT^ method after the data were normalized to *Lt18s*. All the values are shown as the mean ± standard error of the mean, using at least three biological replicates.

### 4.11. Statistical Analysis

All data were measured using at least three biological replicates, and the experiments were repeated at least three times independently. Data were collated using Excel, statistically analyzed using SPSS 22.0 (IBM, Chicago, IL, USA), analyzed for significance using one-way ANOVA (*, *p* < 0.05; **, *p* < 0.01), and plotted using GraphPad 8.0 (GraphPad Software, San Diego, CA, USA) software.

## 5. Conclusions

Duckweed is well known for its potential as feedstock for bioenergy. This is not only because it is an aquatic plant, meaning that it avoids competition for limited arable land; more importantly, the duckweed plant can accumulate starch under nitrogen starvation conditions, which provides a unique advantage over other crops. This work reported, for the first time, that over-expression of *AtPSAT1* in duckweed can facilitate its growth and prompt starch synthesis under nitrogen starvation conditions. The better growth and elevated photosynthesis capabilities were due to up-regulation of the expression of key genes involved in nitrogen assimilation in the *AtPSAT1* over-expressing lines under nitrogen starvation. The high starch accumulation was due to the regulation of expression of genes involved in the glycolytic and TCA cycle pathways, as well as genes involved in the starch synthesis and metabolism pathway. This study provides a prospective pathway for the accumulation of starch through genetically engineering the PPSB pathway into the plant, and sheds light on the mechanism by which PPSB promotes starch accumulation under nitrogen starvation.

## Data Availability

Not applicable.

## Supplementary Materials

The following supporting information can be downloaded at: https://www.mdpi.com/article/10.3390/ijms231911563/s1.

## References

[1] Xu J., Zhao H., Stomp A.-M., Cheng J.J. (2012). The Production of Duckweed as a Source of Biofuels. Biofuels.

[2] Souto L.R.F., da Silva I.F., Ninow J.L., Collins S.R., Elliston A., Waldron K.W. (2019). Effect of Hydrothermal Pre-Treatment on Duckweed (*Landoltia punctata*) Biomass for Simultaneous Saccharification and Fermentation Process. Biomass Bioenergy.

[3] Reid M.S., Bieleski R.L. (1970). Response of *Spirodela oligorrhiza* to Phosphorus Deficiency. Plant Physiol..

[4] Mohedano R.A., Costa R.H.R., Filho P.B. (2016). Effects of CO_2_ Concentration on Nutrient Uptake and Starch Accumulation by Duckweed Used for Wastewater Treatment and Bioethanol Production. Rev. Latinoam. de Biotecnol. Ambient. Y Algal.

[5] Liu Y., Wang X., Fang Y., Huang M., Chen X., Zhang Y., Zhao H. (2018). The Effects of Photoperiod and Nutrition on Duckweed (*Landoltia punctata*) Growth and Starch Accumulation. Ind. Crop. Prod..

[6] Yin Y., Yu C., Yu L., Zhao J., Sun C., Ma Y., Zhou G. (2015). The Influence of Light Intensity and Photoperiod on Duckweed Biomass and Starch Accumulation for Bioethanol Production. Bioresour. Technol..

[7] Liu Y., Chen X., Wang X., Fang Y., Zhang Y., Huang M., Zhao H. (2019). The Influence of Different Plant Hormones on Biomass and Starch Accumulation of Duckweed: A Renewable Feedstock for Bioethanol Production. Renew. Energy.

[8] McLaren J.S., Smith H. (1976). The Effect of Abscisic Acid on Growth, Photosynthetic Rate and Carbohydrate Metabolism in *Lemna minor* L.. New Phytol..

[9] McCombs P.J.A., Ralph R.K. (1972). Protein, Nucleic Acid and Starch Metabolism in the Duckweed *Spirodela Oligorrhiza*, Treated with cytokinins. Biochem. J..

[10] Wang X., Cui W., Hu W., Feng C. (2020). Abscisic Acid-Enhanced Starch Accumulation of Bioenergy Crop Duckweed (*Spirodela polyrrhiza*). RSC Adv..

[11] Faizal A., Putra R.T. (2019). Uniconazole Increases Starch Content in Duckweed (*Lemna aequinoctialis* Welw.). 3BIO J. Biol. Sci. Technol. Manag..

[12] Zhu Y., Li X., Gao X., Sun J., Ji X., Feng G., Shen G., Xiang B., Wang Y. (2021). Molecular Mechanism Underlying the Effect of Maleic Hydrazide Treatment on Starch Accumulation in S. *Polyrrhiza* 7498 Fronds. Biotechnol. Biofuels.

[13] Tao X., Fang Y., Xiao Y., Jin Y.-L., Ma X.-R., Zhao Y., He K.-Z., Zhao H., Wang H.-Y. (2013). Comparative Transcriptome Analysis to Investigate the High Starch Accumulation of Duckweed (*Landoltia punctata*) Under Nutrient Starvation. Biotechnol. Biofuels.

[14] Li J.-M., Du A.-P., Liu P.-H., Tian X.-P., Jin Y.-L., Yi Z.-L., He K.-Z., Fang Y., Zhao H. (2021). High Starch Accumulation Mechanism and Phosphorus Utilization Efficiency of Duckweed (*Landoltia punctata*) Under Phosphate Starvation. Ind. Crop. Prod..

[15] Sun Z., Guo W., Zhao X., Yang J., Duan P., Xu S., Hou H. (2021). Sulfur Limitation Increases Duckweed Starch Accumulation Without Compromising Growth. Biorxiv.

[16] Yu C., Zhao X., Qi G., Bai Z., Wang Y., Wang S., Ma Y., Liu Q., Shumin W., Zhou G. (2017). Integrated Analysis of Transcriptome and Metabolites Reveals an Essential Role of Metabolic Flux in Starch Accumulation Under Nitrogen Starvation in Duckweed. Biotechnol. Biofuels.

[17] Guo L., Jin Y., Xiao Y., Tan L., Tian X., Ding Y., He K., Du A., Li J., Yi Z. (2019). Energy-Efficient and Environmentally Friendly Production of Starch-Rich Duckweed Biomass Using Nitrogen-Limited Cultivation. J. Clean. Prod..

[18] Ros R., Muñoz-Bertomeu J., Krueger S. (2014). Serine in Plants: Biosynthesis, Metabolism, and Functions. Trends Plant Sci..

[19] Ho C.-L., Saito K. (2001). Molecular Biology of The Plastidic Phosphorylated Serine Biosynthetic Pathway in *Arabidopsis thaliana*. Amino Acids.

[20] Cascales-Miñana B., Muñoz-Bertomeu J., Flores-Tornero M., Anoman A.D., Pertusa J., Alaiz M., Osorio S., Fernie A.R., Segura J., Ros R. (2013). The Phosphorylated Pathway of Serine Biosynthesis Is Essential Both for Male Gametophyte and Embryo Development and for Root Growth in Arabidopsis. Plant Cell.

[21] Zimmermann S.E., Benstein R.M., Flores-Tornero M., Blau S., Anoman A.D., Rosa-Téllez S., Gerlich S.C., A Salem M., Alseekh S., Kopriva S. (2021). The Phosphorylated Pathway of Serine Biosynthesis Links Plant Growth with Nitrogen Metabolism. Plant Physiol..

[22] Wulfert S., Krueger S. (2018). *Phosphoserine Aminotransferase1* Is Part of the Phosphorylated Pathways for Serine Biosynthesis and Essential for Light and Sugar-Dependent Growth Promotion. Front. Plant Sci..

[23] Igamberdiev A.U., Kleczkowski L.A. (2018). The Glycerate and Phosphorylated Pathways of Serine Synthesis in Plants: The Branches of Plant Glycolysis Linking Carbon and Nitrogen Metabolism. Front. Plant Sci..

[24] Benstein R.M., Ludewig K., Wulfert S., Wittek S., Gigolashvili T., Frerigmann H., Gierth M., Flügge U.-I., Krueger S. (2013). *Arabidopsis* Phosphoglycerate Dehydrogenase1 of the Phosphoserine Pathway Is Essential for Development and Required for Ammonium Assimilation and Tryptophan Biosynthesis. Plant Cell.

[25] Ho C.-L., Noji M., Saito M., Yamazaki M., Saito K. (1998). Molecular Characterization of Plastidic Phosphoserine Aminotrans-Ferase in Serine Biosynthesis from Arabidopsis. Plant J. Cell Mol. Biol..

[26] Ho C.-L., Noji M., Saito K. (1999). Plastidic Pathway of Serine Biosynthesis—Molecular Cloning and Expression of 3-Phosphoserine Phosphatase from *Arabidopsis thaliana*. J. Biol. Chem..

[27] Sekula B., Ruszkowski M., Dauter Z. (2018). Structural Analysis of Phosphoserine Aminotransferase (Isoform 1) from *Arabidopsis thaliana*—The Enzyme Involved in the Phosphorylated Pathway of Serine Biosynthesis. Front. Plant Sci..

[28] Zhao Z., Shi H.-J., Wang M.-L., Cui L., Zhao H., Zhao Y. (2015). Effect of Nitrogen and Phosphorus Deficiency on Transcriptional Regulation of Genes Encoding Key Enzymes of Starch Metabolism in Duckweed (*Landoltia punctata*). Plant Physiol. Biochem..

[29] Masclaux-Daubresse C., Reisdorf-Cren M., Pageau K., Lelandais M., Grandjean O., Kronenberger J., Valadier M.-H., Feraud M., Jouglet T., Suzuki A. (2006). Glutamine Synthetase-Glutamate Synthase Pathway and Glutamate Dehydrogenase Play Distinct Roles in the Sink-Source Nitrogen Cycle in Tobacco. Plant Physiol..

[30] Sulpice R., Pyl E.-T., Ishihara H., Trenkamp S., Steinfath M., Witucka-Wall H., Gibon Y., Usadel B., Poree F., Piques M.C. (2009). Starch as a Major Integrator in the Regulation of Plant Growth. Proc. Natl. Acad. Sci. USA.

[31] MacNeill G.J., Mehrpouyan S., Minow M.A.A., Patterson J.A., Tetlow I.J., Emes M.J. (2017). Starch as a source, starch as a sink: The bifunctional role of starch in carbon allocation. J. Exp. Bot..

[32] Qu J., Xu S., Zhang Z., Chen G., Zhong Y., Liu L., Zhang R., Xue J., Guo D. (2018). Evolutionary, structural and expression analysis of core genes involved in starch synthesis. Sci. Rep..

[33] Scheidig A., Frohlich A., Schulze S., Lloyd J.R., Kossmann J. (2002). Downregulation of a Chloroplast-Targeted Beta-Amylase Leads to a Starch-Excess Phenotype in Leaves. Plant J. Cell Mol. Biol..

[34] Critchley J.H., Zeeman S., Takaha T., Smith A.M., Smith S. (2001). A Critical Role for Disproportionating Enzyme in Starch Breakdown Is Revealed by A Knock-Out Mutation in Arabidopsis. Plant J. Cell Mol. Biol..

[35] Caballero L., Bancel E., Debiton C., Branlard G. (2008). Granule-Bound Starch Synthase (GBSS) Diversity of Ancient Wheat and Related Species. Plant Breed..

[36] Smith A.M. (2012). Starch in the Arabidopsis Plant. Starch Starke.

[37] Reddy M.M., Ulaganathan K. (2015). Nitrogen Nutrition, Its Regulation and Biotechnological Approaches to Improve Crop Productivity. Am. J. Plant Sci..

[38] Zhang Z., Xiong S., Wei Y., Meng X., Wang X., Ma X. (2017). The Role of Glutamine Synthetase Isozymes in Enhancing Nitrogen Use Efficiency of N-Efficient Winter Wheat. Sci. Rep..

[39] Bernard S.M., Habash D. (2009). The Importance of Cytosolic Glutamine Synthetase in Nitrogen Assimilation and Recycling. New Phytol..

[40] Dellero Y. (2020). Manipulating Amino Acid Metabolism to Improve Crop Nitrogen Use Efficiency for a Sustainable Agriculture. Front. Plant Sci..

[41] Avin-Wittenberg T., Baluška F., Bozhkov P., Elander P.H., Fernie A.R., Galili G., Hassan A., Hofius D., Isono E., Le Bars R. (2018). Autophagy-related approaches for improving nutrient use efficiency and crop yield protection. J. Exp. Bot..

[42] Thomsen H.C., Eriksson D., Møller I.S., Schjoerring J.K. (2014). Cytosolic Glutamine Synthetase: A Target for Improvement of Crop Nitrogen Use Efficiency?. Trends Plant Sci..

[43] Lan S., Wu L., Zhang D., Hu C., Liu Y. (2010). Effects of Drought and Salt Stresses on Man-Made Cyanobacterial Crusts. Eur. J. Soil Biol..

[44] Wang W., Messing J. (2012). Analysis of ADP-Glucose Pyrophosphorylase Expression During Turion Formation Induced by Abscisic Acid in *Spirodela polyrhiza* (Greater Duckweed). BMC Plant Biol..

[45] Basurko M.-J., Marche M., Darriet M., Cassaigne A. (1999). Phosphoserine Aminotransferase, the Second Step-Catalyzing Enzyme for Serine Biosynthesis. IUBMB Life.

[46] Armand D.A., Muñoz-Bertomeu J., Rosa-Téllez S., Flores-Tornero M., Serrano R., Bueso E., Alisdair R.F., Segura J., Ros R. (2015). Plastidial Glycolytic Glyceraldehyde-3-Phosphate Dehydrogenase is an Important Determinant in the Carbon and Nitrogen Metabolism of Heterotrophic cells in Arabidopsis. Plant Physiol..

[47] Wang Y., Kandeler R. (1994). Promotion of Flowering by a Tumor Promoter. J. Plant Physiol..

[48] Yang L., Han Y., Wu D., Yong W., Liu M., Wang S., Liu W., Lu M., Wei Y., Sun J. (2017). Salt and Cadmium Stress Tolerance Caused by Overexpression of the *Glycine Max* Na^+^/H^+^ Antiporter (*Gmnhx1*) Gene in Duckweed (*Lemna turionifera* 5511). Aquat. Toxicol..

[49] Su C., Jiang Y., Yang Y., Zhang W., Xu Q. (2018). Responses of Duckweed (*Lemna minor* L.) to Aluminum Stress: Physiological and Proteomics Analyses. Ecotoxicol. Environ. Saf..

[50] Ziegler P., Adelmann K., Zimmer S., Schmidt C., Appenroth K.-J. (2014). Relative In Vitro Growth Rates of Duckweeds (*Lemnaceae*) —The Most Rapidly Growing Higher Plants. Plant Biol..

[51] Arnon D.I. (1949). Copper Enzymes in Isolated Chloroplasts. Polyphenoloxidase in *Beta vulgaris*. Plant Physiol..

