# Supplementary material for: Over-Expression of Phosphoserine Aminotransferase-Encoding Gene (AtPSAT1) Prompts Starch Accumulation in L. turionifera under Nitrogen Starvation
Source: Int J Mol Sci. 2022 Sep 30;23(19):11563. doi: 10.3390/ijms231911563 (PMC9570139; doi:10.3390/ijms231911563)
Supplement: Supplementary file 1 [file ijms-23-11563-s001.zip › supplementary file.pdf]

Supplementary Materials

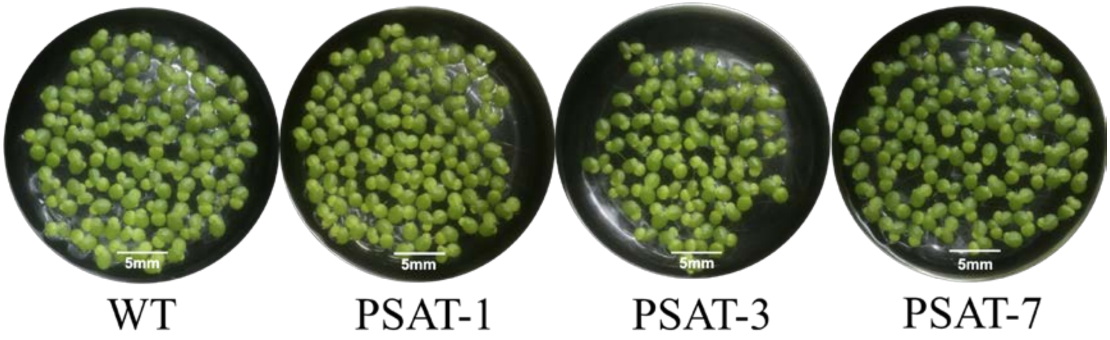

Figure S1. Phenotypes of WT and three *AtPSAT1* transgenic lines. The photoes were taken after WT and *AtPSAT1* transgenic plants were cultivated under full nutrition condition (Datko) for 9 days.

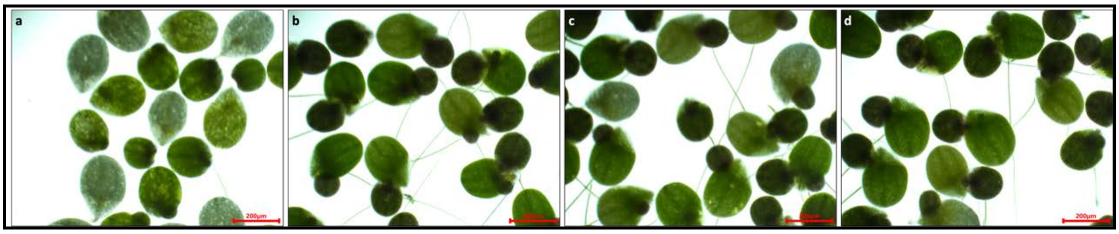

Figure S2. Phenotypes of WT and three *AtPSAT1* transgenic lines under nitrogen starvation condition. **(a)** WT; **(b)** PSAT-1; **(c)** PSAT-3; **(d)** PSAT-7.The photoes were taken after WT and *AtPSAT1* transgenic plants were cultivated under nitrogen starvation condition for 9 days with dissecting microscope.

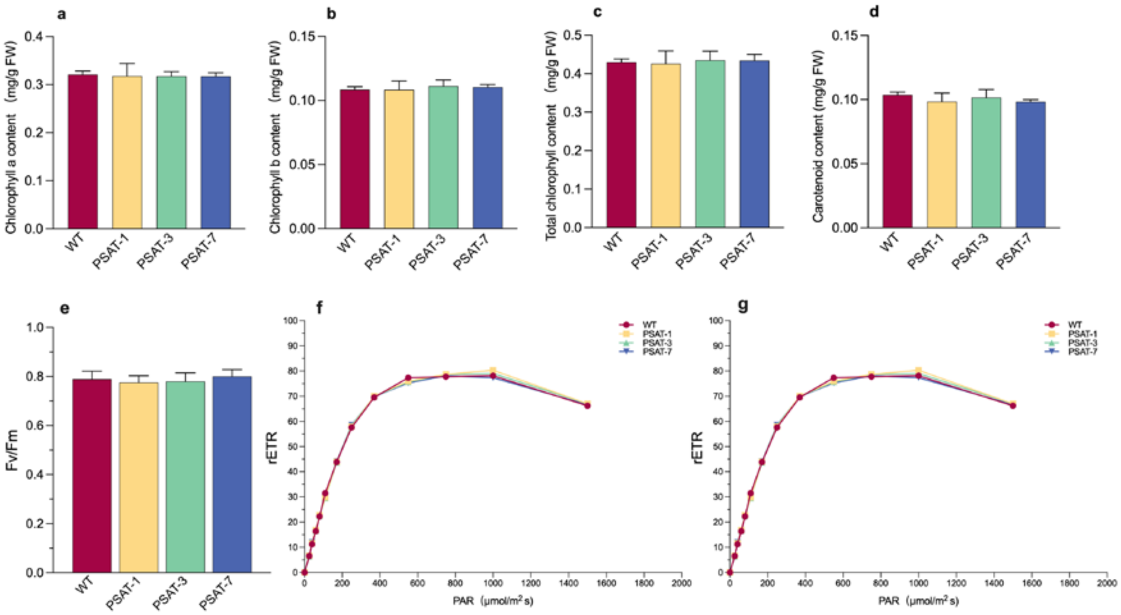

Figure S3. The analysis of photosynthetic pigment and chlorophyll fluorescence-related parameters in duckweed under full nutrition condition (Datko). (a) Content of Chlorophyll a ; (b) Content of Chlorophyll b; (c) Total chlorophyll content; (d) Carotenoid content. The content of photosynthetic pigment were measured after the samples were cultivated under full nutrition condition for 9 days; (e) Value of Fv/Fm; (f) The value of rETR; (g) The value of Y(II), chlorophyll fluorescence-related parameters were measured after the samples were cultivated under full nutrition condition for 6 days. Values given are means ± standard error (n=3) (the data no average value in Figure f and g). The asterisk symbol (*) represents statistically significant differences ($p < 0.05$), double asterisk symbol(**) indicates the difference to the control is significant based on One-way ANOVA ($p < 0.01$).

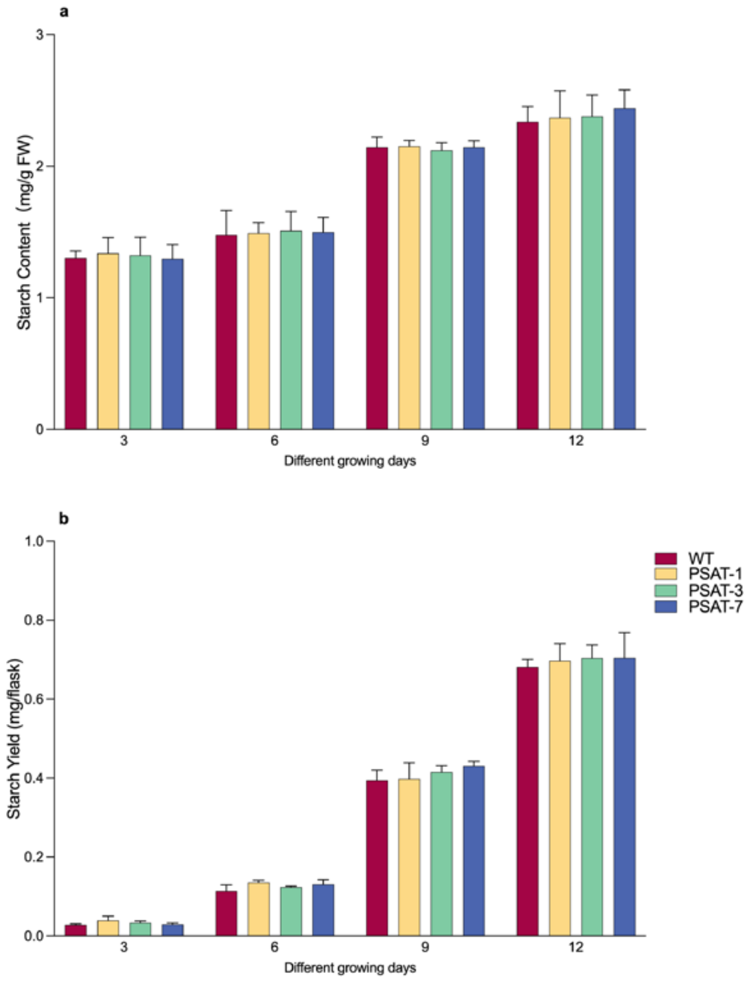

Figure S4. The starch content and yield of WT and three *AtPSAT1* transgenic lines under full nutrition condition in different growing days. **(a)** The starch content; **(b)** The starch yield. Values given are means ± SE of at least 3 independent biological repeats. The asterisk symbol (*) represents statistically significant differences ($p < 0.05$), double asterisk symbol (**) indicates statistically highly significant differences ($p < 0.01$), based on One-way ANOVA .

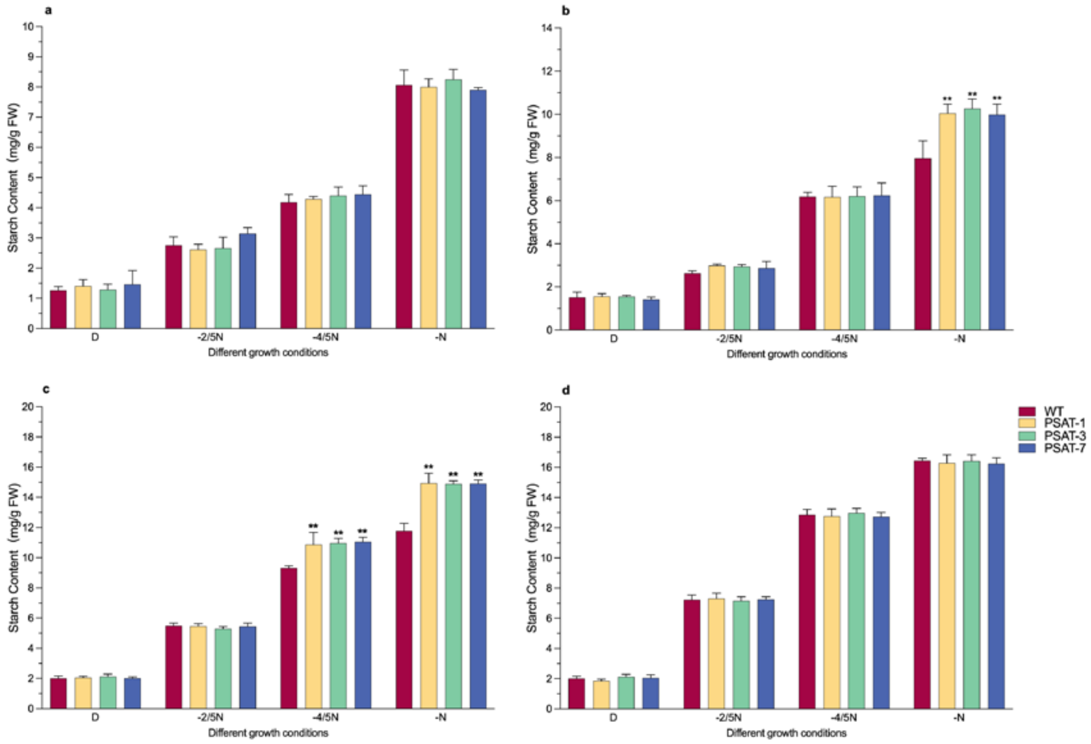

Figure S5. The starch content of WT and three *AtPSAT1* transgenic lines under different nitrogen content culture condition in different growing days. **(a)** The starch content of 3 days; **(b)** The starch content of 6 days; **(c)** The starch content of 9 days; **(d)** The starch content of 12 days. D is full nutrient conditions, -2/5N is lack of 2/5 nitrogen in culture medium, -4/5N is lack of 4/5 nitrogen in culture medium, -N is lack of the whole nitrogen in culture medium. Values given are means ± SE of at least 3 independent biological repeats. The asterisk symbol (*) represents statistically significant differences ($p < 0.05$), double asterisk symbol (**) indicates statistically highly significant differences ($p < 0.01$), based on ne-way ANOVA .

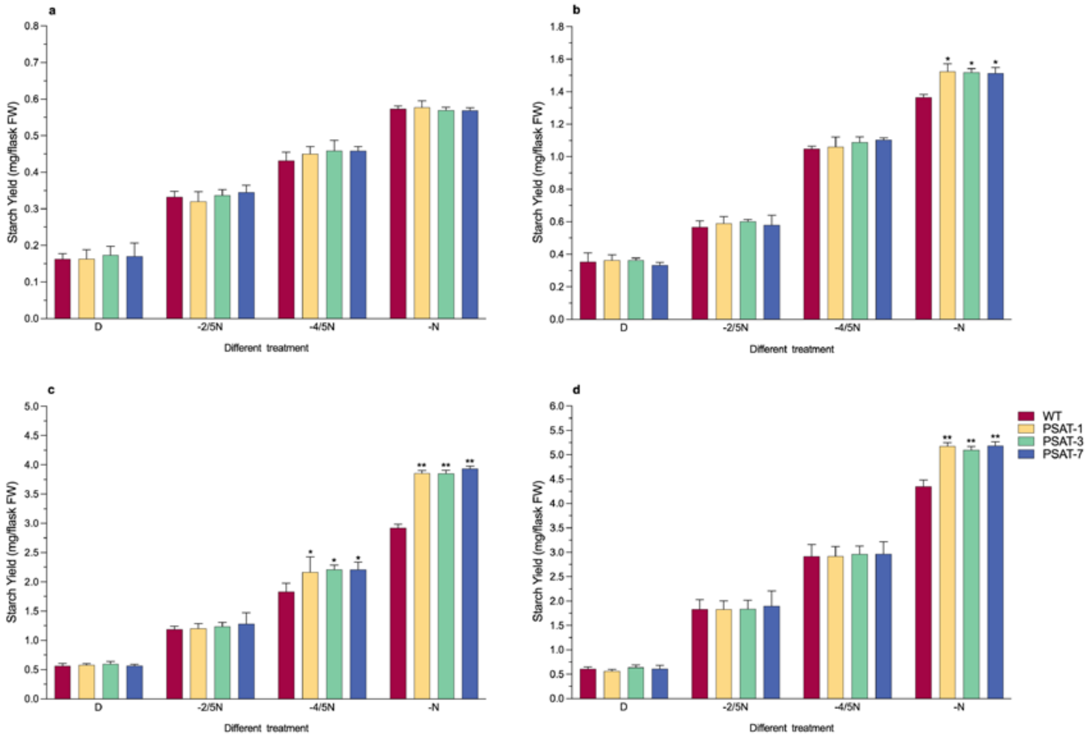

Figure S6. The starch yield of WT and three *AtPSAT1* transgenic lines under different nitrogen content culture condition in different growing days. **(a)** The starch yield of 3 days ; **(b)** The starch yield of 6 days; **(c)** The starch yield of 9 days; **(d)** The starch yield of 12 days. D is full nutrient conditions, -2/5N is lack of 2/5 nitrogen in culture medium, -4/5N is lack of 4/5 nitrogen in culture medium, -N is lack of the whole nitrogen in culture medium. Values given are means ± SE of at least 3 independent biological repeats. The asterisk symbol (*) represents statistically significant differences ($p < 0.05$), double asterisk symbol (**) indicates statistically highly significant differences ($p < 0.01$), based on One-way ANOVA .

Table S1 The primer sequences in the study.

**Primer name**	**Primer sequence（5′-3′）**	**Note**
AtPSAT1-F	CCATGGGGATGGCGGCTACGACGAACT	The primers for genes amplification
AtPSAT1-R	GGGTAACCCCTAAGCATGCTTAGCCTGG	
AtPSAT1-RT-F	TCGGAAGCAACAACACTC	The primers of genes for RT-PCR
AtPSAT1-RT-R	GAAGCGACGCATCTAACA	
Lt18S-RT-F	ATACCGTCCTAGTCTCAACCA	
Lt18S-RT-R	ACAAATCGCTCCACCAAC	
AtPSAT1-qRT-F	GTTGGTCCATCTGGTGTC	
AtPSAT1-qRT-R	CAAAGCAAGGAGGCGTGT	
Lt18S-qRT-F	ATAAACGATGCCGACCAG	
Lt18S-qRT-R	TCAGCCTTGCGACCATAC	

LtNR1-qRT-F	GGTGCTCAAAGACCCAGAA	
LtNR1-qRT-R	TCCGTGATGTACCCAACG	
LtNIR1-qRT-F	GGTTGATGGGTTGTATGGC	
LtNIR1-qRT-R	TCCTCATCCTCCTCCTTTCT	
LtGLN1-qRT-F	CGGAAGGGACACGGAGAA	
LtGLN1-qRT-R	CCTGCCCAAGGAAGAAGA	
LtGS2-qRT-F	GCTCCAGCACAGGTCAAG	
LtGS2-qRT-R	TGTAGCAGTCGCACATCAC	
LtGLT1-qRT-F	CGTTGACTATGGGCACCA	
LtGLT1-qRT-R	TCTGAGCCAGCGACTTCC	
LtGLU1-qRT-F	CTCCATTTGCCCATTGCT	
LtGLU1-qRT-R	TCCATAGCCGCCTTCCAG	The primers of genes for qRT-PCR
LtASN1-qRT-F	CACCAGTACGACTGCCTGAG	
LtASN1-qRT-R	CGTCATCAAACGCCTTCC	
LtASP1-qRT-F	GATTCCAGCCAGATAGTCAA	
LtASP1-qRT-R	GCACAAGCGTGTAGCAAG	
LtGAD1-qRT-F	GTCGAGCTGAAGGAGGTG	
LtGAD1-qRT-R	GAGGAGGTCATTGAGGAGTT	
LtAPS1-qRT-F	ATAGAGCATAGCGTCGTTG	
LtAPS1-qRT-R	TTCATCCTCCGTCTCATAG	
LtSSS1-qRT-F	TGTGGCTCTTTGCCTGTA	
LtSSS1-qRT-R	TTCAACTTCGTGCTCTGC	
LtGBSS1-qRT-F	CGGGTAACAGGAAGGGTG	
LtGBSS1-qRT-R	TCCGGTTCTTTGGTGATTGA	
LtAPL1-qRT-F	CACCGTATCCCAAGTCCC	
LtAPL1-qRT-R	TCACCGTCTCGAAATCGT	
LtISA1-qRT-F	GCACGCCTCTGAGCAACCCT	
LtISA1-qRT-R	TGCCCATTCCATTCTGACCACA	
Ltα-Amy1-qRT-F	ATAATCCGCTCAGAGTTCG	
Ltα-Amy1-qRT-R	ACCGATACCTTACCCTCC	
Ltβ-Amy1-qRT-F	GGCATTCATTGGTGGTAC	
Ltβ-Amy1-qRT-R	GTCGTGCTTCTTGAGGGT	
LtPFK1-qRT-F	TCGGAATAATCGCTGTCTC	
LtPFK1-qRT-R	CGTGAACTTGTGCCTTACC	
LtFBA1-qRT-F	AACCTCAATGCCATGAACC	
LtFBA1-qRT-R	TCCACCTTACCTGCCCAC	
LtPK1-qRT-F	TCAGGCAGAGCAGCAAAG	
LtPK1-qRT-R	CTGGGTTCCCGAGAAAGT	
LtPDC1-qRT-F	GCCCAATCTTCAACGACTA	The primers of genes for
LtPDC1-qRT-R	CTTCATCAGAACGCACCC	

LtACO1-qRT-F	GGAGTGACCGCAACAGAT	qRT-PCR
LtACO1-qRT-R	GCACCGTATTCAGGAGACA	
LtIDH1-qRT-F	AACGACATACGGCTTCTA	
LtIDH1-qRT-R	TTACCCATTACCTGACAAC	
Lt2OG-DH1-qRT-F	ATTCACGGAGATGGGAGTT	
Lt2OG-DH1-qRT-R	CAGCGGTCGGATCAGTAG	
LtSDH1-qRT-F	AATGTAACCAAGGAGCCG	
LtSDH1-qRT-R	TTTGCGCCATGAACTGAT	
LtFUM1-qRT-F	AACGACATACGGCTTCTA	
LtFUM1-qRT-R	ATTACCCATTACCTGACAAC	
LtMDH1-qRT-F	GCTTCATCTGGGTCTGGT	
LtMDH1-qRT-R	CGTTCGTCATCGTCGTTA	
LtCS1-qRT-F	TTGGACTATGGCGGAAAT	
LtCS1-qRT-R	GCAGTATGGGCGCTAACA	
